# Recent advances in microbial engineering approaches for wastewater treatment: a review

**DOI:** 10.1080/21655979.2023.2184518

**Published:** 2023-07-27

**Authors:** Monika Sharma, Sangita Agarwal, Richa Agarwal Malik, Gaurav Kumar, Dan Bahadur Pal, Mamun Mandal, Abhijit Sarkar, Farkad Bantun, Shafiul Haque, Pardeep Singh, Neha Srivastava, Vijai Kumar Gupta

**Affiliations:** a Department of Zoology, University of Jammu, Jammu and Kashmir, India; b Department of Applied Science, RCC Institute of Information Technology Kolkata, West Bengal, India; c Department of Environmental Studies, PGDAV College, University of Delhi, New Delhi, India; d Department of Chemical Engineering, Harcourt Butler Technical University, Kanpur, Uttar Pradesh, India; e Laboratory of Applied Stress Biology, Department of Botany, University of Gour Banga, Malda, West Bengal, India; f Department of Microbiology, Faculty of Medicine, Umm Al-Qura University, Makkah, Saudi Arabia; g Research and Scientific Studies Unit, College of Nursing and Allied Health Sciences, Jazan University, Jazan, Saudi Arabia; h Department of Chemical Engineering and Technology, Indian Institute of Technology (BHU), Varanasi, India; i Biorefining and Advanced Materials Research Center, SRUC, UK; j Gilbert and Rose-Marie Chagoury School of Medicine, Lebanese American University, Beirut, Lebanon; k Centre of Medical and Bio-Allied Health Sciences Research, Ajman University, Ajman, United Arab Emirates

**Keywords:** Wastewater treatment, bacteria, fungi, yeast, microalgae, microbial consortium, organic pollutant, inorganic pollutant

## Abstract

In the present era of global climate change, the scarcity of potable water is increasing both due to natural and anthropogenic causes. Water is the elixir of life, and its usage has risen significantly due to escalating economic activities, widespread urbanization, and industrialization. The increasing water scarcity and rising contamination have compelled, scientists and researchers, to adopt feasible and sustainable wastewater treatment methods in meeting the growing demand for freshwater. Presently, various waste treatment technologies are adopted across the globe, such as physical, chemical, and biological treatment processes. There is a need to replace these technologies with sustainable and green technology that encourages the use of microorganisms since they have proven to be more effective in water treatment processes. The present review article is focused on demonstrating how effectively various microbes can be used in wastewater treatment to achieve environmental sustainability and economic feasibility. The microbial consortium used for water treatment offers many advantages over pure culture. There is an urgent need to develop hybrid treatment technology for the effective remediation of various organic and inorganic pollutants from wastewater.

## HIGHLIGHTS

- Microbial engineering approaches for wastewater treatment.
- Current and emerging sources of water pollution are discussed.
- Various treatment technologies for wastewater treatment.
- Biological methods and microbes are used for degradation.
- Parameters responsible for the degradations processes of wastewater.

## GRAPHICAL ABSTRACT

**Figure:**
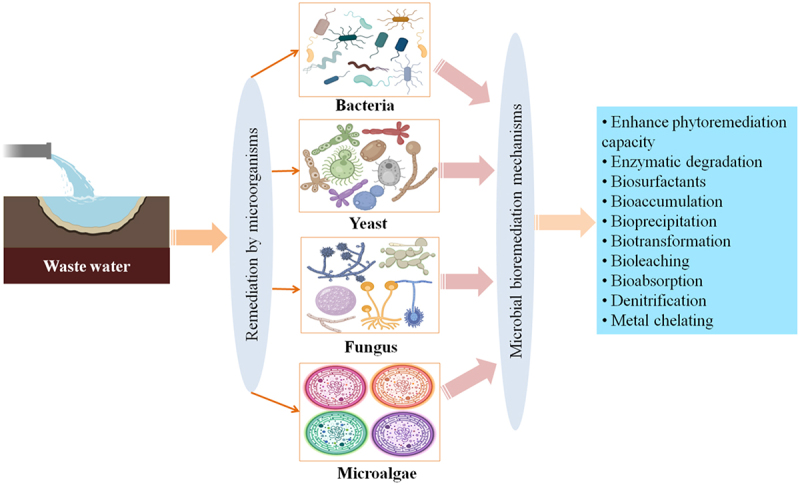

## 1 Introduction

World water consumption has almost doubled in a few decades [1]. The growing concerns over water contamination have led to extensive research and development in water treatment techniques. They are expanding to promote the reuse of water and improve the quality of water for human consumption. Water pollution is a worldwide issue that presents a severe threat to the survival of all life forms. Aquatic pollution can be caused by organic and inorganic impurities and microbiological contaminants. Population growth, industrial and mining activities, sewage and wastewater, radioactive waste, chemical fertilizers, pesticides, urban development, and other anthropogenic sources are all responsible for rising levels of aquatic pollution. Water quality is determined by the concentrations of particles and chemicals in water, such as heavy metals, nutrients, microorganisms, polycyclic aromatic hydrocarbon (PAH), and other pollutants. Many organic contaminants are endocrine-disrupting chemicals associated with testicular, prostate, and breast cancers. They can also cause serious complications in human and animal reproductive health, such as sperm count reduction in males and the production of fragile eggs in females, among other things [2].

Wastewater treatment has evolved as a feasible technique for tackling water scarcity and protecting the ecosystem from the harmful impacts of polluted/wastewater in the contemporary environment [3]. Many countries have passed stricter laws for treating sewage water before dumping it into water bodies. From sustainability, improved water management and wastewater recycling have begun to get active attention [4]. Many physical and chemical processes (flotation, precipitation, oxidation, adsorption, etc.) used for wastewater treatment are expensive, demand high maintenance, and require a complicated functional setup. As a result, there is an urgent necessity to move to green and sustainable technologies such as microbial wastewater treatment to serve as a long-term alternative to traditional wastewater treatment methods [3]. Wastewater treatment using microbes such as fungi, bacteria, microalgae, and others has caught the researcher’s attention in recent years. The presence of a significant amount of nutrients such as nitrogen, phosphorus, and carbon in wastewater emanating from different sources can aid in the growth and survival of these microbes [5].

Phytoremediation is frequently used to remediate wastewater. On the other hand, excessive salts in wastewater can induce plant toxicity. To solve the problem, Sarawaneeyaruk et al. [6] isolated multifarious plant growth-promoting bacteria (PGPB) *Bacillus*spp from the municipal wastewater and by using this PGPB they successfully enhanced plant growth under municipal wastewater irrigation. Hence, such green technology would be sustainable and help maintain a balance between socio-economic and environmental perspectives [3].

Food, water, and energy are interconnected and wastewater is crucial in this nexus. Although various wastewater treatment methods are available the aim of this review is to draw attention on the importance of microbes in wastewater. The goal of this review is to help in protecting water resources using effective treatment method. This review illustrates the wastewater treatment process by utilizing microorganisms (bacteria, fungi, microalgae, and yeast), highlighting the advantages and applications of microbes over other conventional approaches. This review further aims to provide useful information to researchers working in relevant fields worldwide to pique their interest in using microbes to improve and cost-effectively treat wastewater (Table 1).

**Table 1.** Categories of water pollutants and their probable effects are tabulated as.

Type of pollutant	Source of origin	Effect on environment
Sediments	Excavation, mining operations, suspended solids	Light penetration into the water becomes limited. Suffocate river bed creatures
Organic components	Any living source (animal, human, plant)	Anaerobic conditions thus cause suffocation and death of aquatic organisms [11] Uncomfortable symptoms, and odour
Nutrients	Surface runoff	Enrichment leads to algal blooms/eutrophication of water bodies [12]
Heavy metals and pesticides	Industrial, municipal and agricultural discharge	Severe health impacts on the living organism as they can be carcinogenic and kill weeds and other aquatic life.
Oil spillage	Disaster or leaks	The surface film cuts off oxygen. Suffocated environment
Biological pollutants Virus	Untreated effluent dumped into a river; from a contaminated person’s open defecation	Water born viral diseases, e.g. Hepatitis A, Polio, Rota virus [13]
Helminthes	Organic waste discharge	Diseases in animals, e.g. Dracunculiasis.
Protozoans	Organic human waste discharge	Animal diseases

## 2 Emerging sources of water pollution

A variety of organic contaminants can be found in water, including insecticides, herbicides and organohalides [7]. Industrial effluents contain inorganic pollutants such as silt from stormwater runoff and heavy metals from acid mine drainage. Different sources of domestic waste enter water bodies. Pesticides used in gardens and lawns may also enter the water bodies [8]. Cleaning products, detergents, and skincare products contain significant amounts of pollutants that can pollute water bodies and make them unfit for human consumption [9]. Chemicals and acids from industries like steel and paper are discharged into rivers [10]. Water bodies receive over 70% of industrial waste, containing many toxins [8]. Major agricultural wastes are fertilizers, pesticides (herbicides, insecticides), and other agrochemicals. Fertilizer production continues to rise year after year to increase productivity, resulting in increased waste generation. Irrigation contributes significantly to surface water pollution in China and is also a cause of nitrogen groundwater pollution in the United States [9]. Toxic chemicals can accumulate in the body and eventually reach toxic levels, causing food chain disruption. Another major source of water pollution is nutrient enrichment.

## 3 Emerging technologies for wastewater treatment

Physical treatment of wastewater entails removing contaminants from the water without affecting the biochemical properties of contaminants. Physical treatments usually follow chemical and biological treatments. Screening, Flotation, Flow equalization, Membrane-based technology, Thermal treatment, and other physical treatment techniques are commonly used. Some of the common chemical unit processes used in wastewater treatment technology include precipitation, adsorption, disinfection, chlorination, neutralization, chemical exchange, etc. [14] to bring changes in the quality of water.

### 3.1 Biological treatment for the wastewater

Biological treatment involves removing contaminants from wastewater using biological organisms or processes. Microbes are critical to wastewater treatment and reclamation, making them a promising green technology tool. The biological treatment uses bacteria, fungi, microalgae, yeast, and other microbial groups. Biological treatments are less expensive than physical and chemical treatments Table 2 [15]. Among the most widely used biological wastewater treatment methods are:

**Table 2.** Showing advantages of biological methods in wastewater treatment [17].

Biological method	Advantages	Characteristic
Bioreactors	Easy and simple	Use of pure and mixed microbial culture
Biological Activated sludge	Simple, economically attractive, and well accepted by the public.	
Microbial Consortiums	A large number of species (in mixed culture) can be used. Efficiently eliminates biodegradable organic matter, NH_4_+, heavy metals etc. Attenuates color well.	
Enzymatic degradation	High removal of BOD and suspended solids. Microbial consortium effectively eliminates pollutants from wastewater.	

#### 3.1.1 Activated sludge method

One of the most commonly used biological processes in wastewater treatment to date is the activated sludge. It has been effectively used to treat industrial and municipal wastewater. This process uses a biological floc that consists of bacteria and protozoa under aerobic conditions [16]. The basic principle behind all activated sludge processes is that microbes grow within metabolizing organic material where they form clumps. The oxidizable matter is used as food for the microorganisms forming a suspended floc in the wastewater. The aeration/agitation provides the continued oxygen supply. The mixed liquor, which is the mix of wastewater and activated sludge, is allowed to settle down to segregate the activated sludge solids from treated wastewater while a part of the settled activated sludge is returned to the aeration site. The entire activated sludge process comprises interlinked elements such as an aeration tank, source of aeration, clarifier, and a collection system. The biological reaction occurs in an aeration tank fitted with a stirrer for mixing and source of oxygen, which is connected to a tank/clarifier where the settled solids are segregated from treated water along with a collection loop which either returns the activated sludge back to the aeration tank or is removed from the process. The process has high efficiency and can also be used for nutrient removal [18].

The activated sludge process is used in the treatment of industrial wastewater as well as domestic. In spite of having advantages like low operational cost with added treatment efficiency, the major drawback is the generation of excess quantity of waste activated sludge. The organic matter generated by the process needs to be properly treated and managed to reduce the ecological and financial burden [19].

#### 3.1.2 Bioreactors and biofilters

Using physical retention and microbial biodegradation, membrane bioreactors eliminate pollutants from wastewater [20]. Biofilters use biological processes to filter wastewater [21]. It grows on top of the media, which is composed of gravel, sand, and ceramic. For example, a biofilm can contain a microbial (bacterial) community that helps to decompose organic content in water [37]. This process has been extensively used to remove H_2_S from municipal wastewater, according to Zhang et al. [38].

#### 3.1.3 Biosorption

Certain biological molecules naturally can accumulate metals like copper, zinc, nickel, chromium, palladium from wastewater [39]. The process of biosorption is complex and involves various interactive mechanisms like ion-exchange, absorption, precipitation, and complexation through the participation of functional groups like hydroxyl, carbonyl, etc. [40]. It is a reversible process that involves interactions rather than oxidation to bind the biosorbent in an aqueous solution.[41]

The biosorbent is suspended in a contaminant solution (e.g. metal ions). After a while, contaminant-rich biosorbent can be separated. Microbes immobilize on adsorbants to form a biosorbent that captures contaminants [42].

Agricultural waste, microbial biomasses, industrial-by-products offer advantages over chemical methods in terms of efficiency, large abundance and low cost [43]. Factors like pH, concentration of the metals, ionic strength, other pollutants present in wastewater, temperature, etc., effect the process of biosorption [44]

## 4 Different microbial groups in wastewater degradation

Microbial treatment can be used instead of traditional wastewater treatment methods because it is cheaper, more efficient, and more competent [45]. Bioremediation involves bacteria, fungi, microalgae, yeast, etc. [46]. These microbes are responsible for degrading or converting contaminants into lesser harmful products [47]. They have become ideal bioagents of remediation owing to their high surface area-to-volume ratio, small size, and substantial surface area [48]. These microbes use biosorption and bioaccumulation to bioremediate. Adsorption occurs when pollutants (metals) interact with functional groups on the cell surface [49]. Biosorption can use both live and dead biomass. Bioaccumulation involves intracellular and extracellular processes. Toxins bioaccumulate when they are absorbed from the environment. Bioaccumulation uses only living biomass, limiting its reuse, and costing more than biosorption [50].

### 4.1 Bacterial removal of organic and inorganic pollutants

The treatment of wastewater effluents is based on the capability of bacterial cells to concentrate pollutants (metals). The microbial population and xenobiotic content determine the rate of biodegradation. Plants feed rhizosphere microbes’ organic carbon, which helps degrade pollutants. Aquatic plants’ biofilms can degrade organics like phenols, amines, and aliphatic aldehydes [51]. Methanotrophs use methane to obtain carbon and energy and break down various harmful organic compounds [52]. *Eichhornia crassipes* can help clean up eutrophic water by influencing nitrogen production [53]. *Tolypothrixceytonica* and *Anabaena oryzae* have also been shown to be effective in treating industrial wastewater [54]. *Aphanocapsu sp*. and *Plectonema sp*. have the ability to degrade crude oil [55]. Anaerobic bacteria in sewage treatments include sulfate-reducing bacteria like *Desulfovibrio, Desulfotomaculum, Desulfobacter, and Desulfococcus genera* [56].

The factors like abundance, size, growth under controlled conditions and resistance to environmental changes have marked bacteria as important biosorbents [57]. Metal ion biosorption into the cell wall can be active or passive. Passive biosorption occurs in both living and dead/inactive bacterial cells [58]. Active biosorption includes metal ion uptake within living bacterial cells. Metal ion binding involves ion exchange, chelation, complexation, and micro precipitation [59].

#### 4.1.1 Dye degradations

Synthetic dyes have many advantages over natural dyes in terms of color variety, speed of coloration, absorption and water solubility [60], which explains the global dye production of 800,000 tons per year [61]. The impact of textile effluents on the overall health of the aquatic ecosystem is growing in concern as dye demand and production rise. Textile wastewater contains inorganic and organic additives and chemicals [62] as well as dyes [63] in concentrations ranging from 10 to 200 mg/L. In textile industries, the azo dyes (70%) are commonly used because of their low cost and ease of use. Since all dyes do not fix to fabrics during dyeing, unfixed dyes are washed out and found in high concentrations in effluents [64]. Bacterial-assisted dye degradation is nontoxic and can decolorize colored complex dyes. Table 3 lists some studies on bacterial dye degradation.

**Table 3.** Shows the bacterial degradation of dyes and the mechanism involved.

Bacteria	Dye	Mechanism	Reference
*Bacillus sp*	Red HE78	Enzymatic degradation by Azoreductase and Laccase	[22]
*Aeromonas sp.*	Methyl Orange	Enzymatic degradation by Laccase, NADH-DCIP reductase and Azo-reductase	[23]
*Shewanellamarisflavi*	Xylidine ponceau 2 R	Flocculation and Enzymatic degradation	[24]
*Pseudomonas extremorietalis*	Congo Red	Enzymatic degradation by Laccase	[25]
*Aeromonas hydrophila SKK16 and Lysinibacillus sphaericus SK13*	Remazol Yellow F3R, Drimaren Black Joyflix Yellow F3R	Enzymatic degradation by Laccase and Azoreductase	[26]
*Aeromonas hydrophila, Lysinibacillus sphaericus*	Reactive Red 195	Enzymatic degradation by Laccase and Azoreductases	[27]
*Bacillus sp., Staphylococcus aureus*	Bemacron Yellow Bemacron Blue	Adsorption and oxidation	[28]
*Escherichia coli*	Methyl Orange	Enzymatic degradation	[29]
*Aeromonas hydrophila*, *Lysinibacillus sphaericus*	Reactive Red F3B Joyfix Yellow MR Remazol Blue Remazol Red RGB	Enzymatic degradation by Laccase and Azoreductase	[30]
*Micrococcus sp.*	Reactive Red −120	Enzymatic degradation by Laccase and Azoreductase	[31]
*Orskoviapaurometabola*	Acid Red 14	Enzymatic degradation	[32]
*Bacillus sp.*	Ponceau 4 R	Enzymatic degradation by Azoreductase	[33]
*Pseudomonas stutzeri*	Acid Blue 113	Enzymatic degradation by Azoreductase and Laccase	[34]
*Aeromonas hydrophila*	Reactive Yellow F3R Joyfix Red RB Remazol yellow RR	Enzymatic degradation by Laccase, Veratryl alcohol oxidase and Azoreductase	[35]
*Anoxybacillus sp.*	Direct Black G	Enzymatic Azoreductase, Pyruvate Kinase, Quinone reductase	[36]

*Aeromonas hydrophila, Bacillus subtilis, Bacillus cereus* have been studied effectively and have the potential for bioremediation of azo dyes [65]. Under anoxic conditions, *Pseudomonas sp., Pseudomonas luteola, Proteus mirabilis* have the ability to degrade azo dyes [66]. These bacteria utilize oxidoreductive enzymes for dye degradation. Aerobic bacteria use oxygen-catalyzed azoreductase to break the azo bonds [67]. Some bacterial strains degrade dyes in aerobic conditions and use mono and dioxygenase for oxidizing the aromatic ring of organic compounds [68]. Anaerobic bacteria use the enzyme azoreductase to degrade azo dyes. And generally, anaerobic conditions favor decolorization (Chang et al., 2001b). Mostly first-order kinetics is followed with respect to the concentration of the dye in the decolorization reaction and in some; zero-order kinetics is also seen [69]. The oxidoreductive enzymes also are involved in hydroxylation, desulfonation, and deamination. *Pseudomonas aeruginosa* could decolorize various azo dyes [70] and Navitan Fast Blue S5R under aerobic conditions.

#### 4.1.2 Petrochemicals degradation

**Figure 1.**
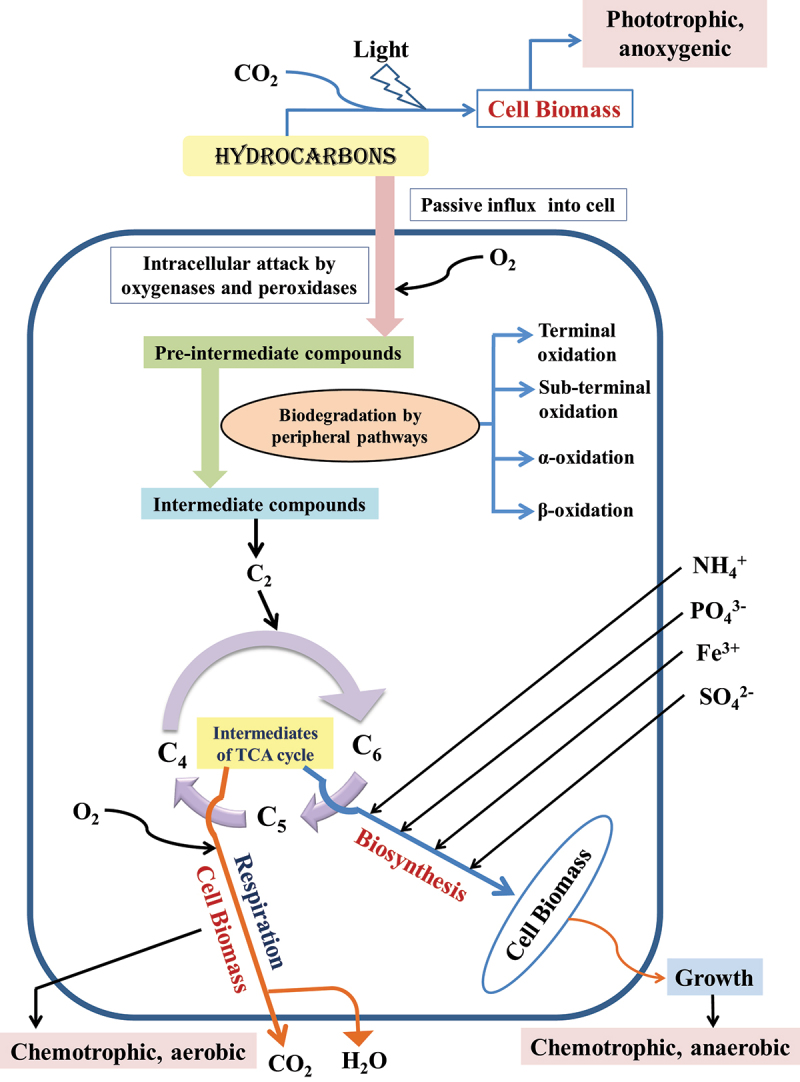
Microorganism mediated degradation of hydrocarbons.

Petroleum hydrocarbons are divided into resins, asphaltenes, aromatics, and saturates [79]. Their degradation by microbes (Figure 1) is complex, dependent on the nature and number of hydrocarbons available. The biodegradation of hydrocarbons is determined by agents such as temperature and concentration of inorganic nutrients such as phosphorus, nitrogen, and iron in some instances [80]. The vulnerability of hydrocarbons to attack by microbes is different, with linear alkanes being most susceptible and cyclic alkanes the least [81,82]. Polycyclic hydrocarbons having higher molecular weight might not be degraded [83]. *Acinetobacter* sp. degrades n-alkane having chain length C_10_-C_40,_ utilizing carbon as the sole source [84]. *Mycobacterium*, *Burkholderia, Gordonia*, *Brevibacterium*, *Dietzia, Aeromicrobium, Pseudomonas, Aeromonas, Flavobacteria, Nocardia, Modococci, Chrobacteria, Moraxella, Cyanobacteria, Streptomyces, Bacilli, Arthrobacter*, and other bacteria can degrade petroleum products [85]. The poly-aromatic hydrocarbons could be degraded by *Sphingomonas* [86]. The biodegradation efficiency of soil bacteria [87] and marine bacteria [88] are not the same. The microbes utilize specific enzymes systems (oxygenase, peroxidase, and hydroxylase) in degrading the petroleum hydrocarbons in aerobic conditions, and it starts with the attachment of microbial cells onto the substrates and is followed by the production of biosurfactants [89,90]. Biosurfactants are synthesized by various microorganisms (Table 4) and are heterogeneous surface-active compounds. Biosurfactants are involved in enhancing the solubility and, finally, the removal of the contaminant [91]. They augment the surface area and the amount of oil available for the bacteria to utilize [92] and decrease surface tension to help form micelles.

**Table 4.** Biosurfactants produced by the various microbes for degradation of petroleum hydrocarbons.

Microorganisms	Biosurfactants	Structure	References
*Pseudomonas aeruginosa, Pseudomonas fluorescens*	Rhamnolipids	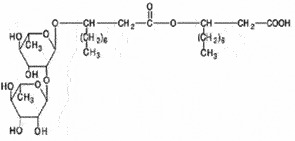	[71] [72]
*Candida tropicalis*	Lipomannan	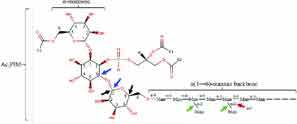	[73]
*Aeromonas*sp., *Bacillus sp.*	Glycolipid	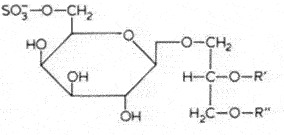	[74; 75]
*Bacillus subtilis*	Surfactin	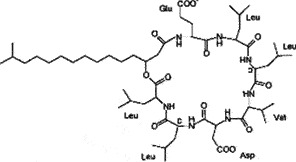	[76]
*Candida bombicola*	Sophorolipids	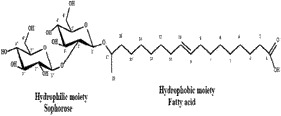	[77, 78–100]

#### 4.1.3 Pharmaceutical and personal care products

**Figure 2.**
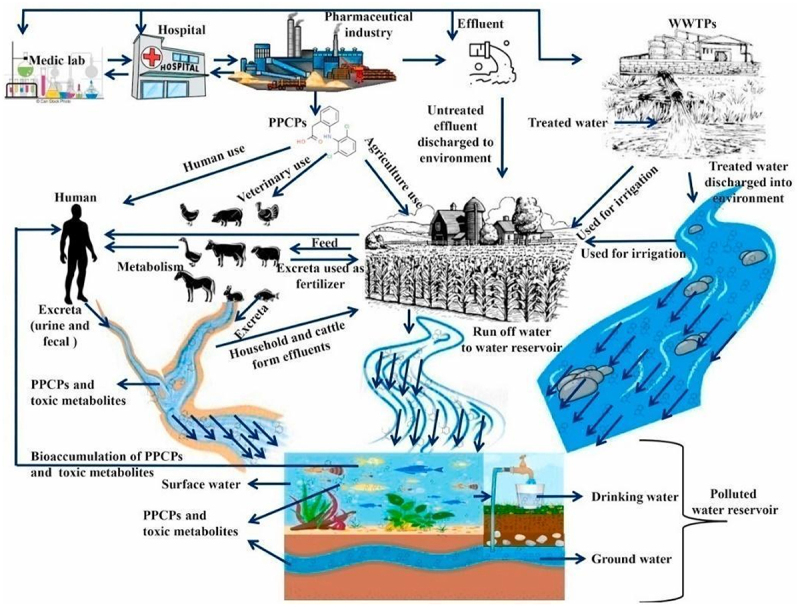
Representative diagram of pharmaceuticals and personal care products circulating in the environment adopted from [73].

Pharmaceuticals and personal care products (PPCPs) are emerging persistent pollutants [93]. Pharmaceuticals have increased steadily globally [94], especially since the COVID-19 pandemic. Individuals contribute PPCPs to the aquatic ecosystem (Figure 2) by using sanitizers, shampoos, household cleaners, detergents, and medicines. PPCPs are complex and persistent molecules that reenter the hydrologic cycle, increasing antibacterial resistance, reproductive abnormalities, and tumor growth. These unregulated pollutants persist in water bodies, and many metabolites are converted back to their parent form [95]. They precipitate specific pollutants into complex and toxic forms that easily spread in aquatic phases. Figure 2 shows the circulation of PPCP in the surrounding [96]. The breakdown of PPCPs by microorganisms is difficult because pharmaceuticals were designed to be toxic to bacteria [97]. Nonetheless, some native bacterial species can help degrade pharmaceutical pollutants [98]. Microbes reduce or degrade the complex structure to a nontoxic or less toxic form.

#### 4.1.4 Pesticides

Pesticides are chemicals utilized to kill pests and are classified based on their functions (herbicides, algicides, fungicides, bactericides, nematicides, rodenticides, and insecticides) [99]. Chemical classes of organic pesticides include organophosphorus, organochlorine, carbamates, acetamides, neonicotinoids, pyrethroids, triazoles, and triazines. Inorganic pesticides include lead arsenate, and boric acid complexes, etc. Organochlorine compounds like chlordane, DDT, toxaphene, and heptachlor have been included in the list of persistent organic pollutants [100,101].

It has been reported by many researchers that effectively, less than 5% of total used pesticides are involved in targeting the pests while the rest of them are precipitated in the surrounding water and soil [102]. The pesticides left in the ecosystem have a detrimental effect on the ecosystem [103] and need to be removed. The chemical and physical methods of pesticide removal are unsustainable [104], hence bacteria could function as bio-weapon to fight toxic agricultural chemicals [105]. Various studies have elaborated on the role of bacteria in bioremediating pesticides, viz. Endosulfan removal by *Bacillus* and *Staphylococcus* [106]; Malathion removal by *Arthrobacter sp*., *Pseudomonas putida* [107]; Ridomil and Fitoraz removal by *Pseudomonas putida* and *Acinetobacter sp*. [108]; Napthelene removal by *Cyanobacteria* [109]; Endosulfan removal by *Staphylococcus aureus*, *Achromobacter sp*., *Rhodococcus sp*. [110]; Malathion, Ridomil and Fitoraz removal by *Pseudomonas putida*, *Rhodococcus* and *Arhtrobacter sp*. [111].

#### 4.1.5 Heavy metals degradation

Heavy metals such as lead (Pb), cadmium (Cd), chromium (Cr), arsenic (As), and mercury (Hg) are ubiquitous environmental pollutants, having high toxicity and density. Natural and anthropogenic sources of heavy metal pollution cause detrimental effects on all living beings [112]. The microbial cells require cations for numerous cellular activities, but increasing concentration may retard growth by forming internal complexes [113]. Bacteria have the ability to immobilize and also mobilize, transform and uptake heavy metals [114]. Many studies have been published on the role of endophytic bacteria in heavy metal bioaccumulation and detoxification [115,116]. These studies show that bacteria secrete organic acids to help with the bioremediation process. Bacteria also produce biosurfactants released as root exudates and increase metal bioavailability in aquatic environments [117]. It was found that glutathione was involved in the intracellular sequestration of cadmium ions in the cells of *Rhizobium leguminosarum* [118]. Heavy metals can be reduced to less or nontoxic metals by iron-reducing bacteria like *Geobacter* sp. and sulfur-reducing bacteria like *Desufuromonas* sp. Sulfate-reducing bacteria and metal-reducing bacteria, for example, can convert chromium from the highly toxic Cr (VI) to the less toxic Cr (III) [119]. Sulfate-reducing bacteria produce a lot of hydrogen sulfide, which causes metal cations to precipitate [120]. *Vibrio harveyi* strain could precipitate the divalent lead as a salt of lead phosphate [121]. Many ionizable cell wall groups can help bacteria absorb metal ions (amino, carboxyl, phosphate, and hydroxyl gp). In metal remediation, microbial methylation is also important. For example, *Bacillus* sp., *Clostridium* sp., *Pseudomonas* sp., and *Escherichia* sp., can biomethylate Hg (II) [122]. Various heavy metals respond to the microorganism differently depending on the conditions. Some bacterial cells produce siderophores, and they form metal complexes, limiting their bioavailability and removing their toxicity [123]. Some of the bacterial species involved in the bioremediation of heavy metals (Table 5) have been tabulated.

**Table 5.** Some Bacterial species used for the removal of heavy metals.

Type of Heavy Metal	Bioremediation using bacterial species	Reference
Cr	*Acinetobacter sp.*	[138]
Cr	*Bacillus sp.*	[139]
Cr	*Pseudomonas aeruginosa*	[140]
Cr	*Cellulosimicrobium sp.(*KX710177)	[141]
Pb	*Bacillus sfirmus*	[142]
Pb	*Staphylococcus sp.*	[143]
Cu, Ni	*Desulfovibriodesulfuricans* KCTC5768 (immobilize on zeolite)	[144]
Cu, Ni	*Micrococcus sp.*	[145]
Co	*Vibriofluvialis*	[146]
Hg	*Enterobacter cloacae*	[147]
Hg	*Klebsiella pneumoniae*	[148]
Hg	*Bacillus licheniformis*	[149]
Zn	*Bacillus sfirmus*	[150]
Zn	*Pseudomonas sp.*	[151]
Mn, Zn, Co	*Acetobacter sp.*	[152]
As	*Herminiimonasarsenicoxydans*	[153]
Cu, Ni, Cr, U	*Pseudomonas aeruginosa, Aeromonas sp.*	[154]
Cd	*Bacillus safensis*	[155]
Pb, Cr, Cd	*Aerococcus sp, Rhodopseudomonas palustris*	[156]
Fe	*Microbacterium profundi*	[157]
Fe, Zn	*Lactobacillus delbrueckii, Streptococcus thermophilus*	[158]
Mn	*Leptothrix, Pseudomonas, Planctomyces*	[159]
Co, Cu, Cr, Pb, Cd	*Lysinibacillus sphaericus, Bacillus safensis*	[160]
Pb	*Ralstonia solanacearum*	[161]
Cd	*Enterobacter aerogenes*	[162]
Mn, Fe, Cu, U, Zn	*Geobacter sp., Pseudomonas fluorescens, Vibrio harveyi, Pseudomonas aeruginosa*	[163]

### 4.2 Fungi and yeast

**Figure 3.**
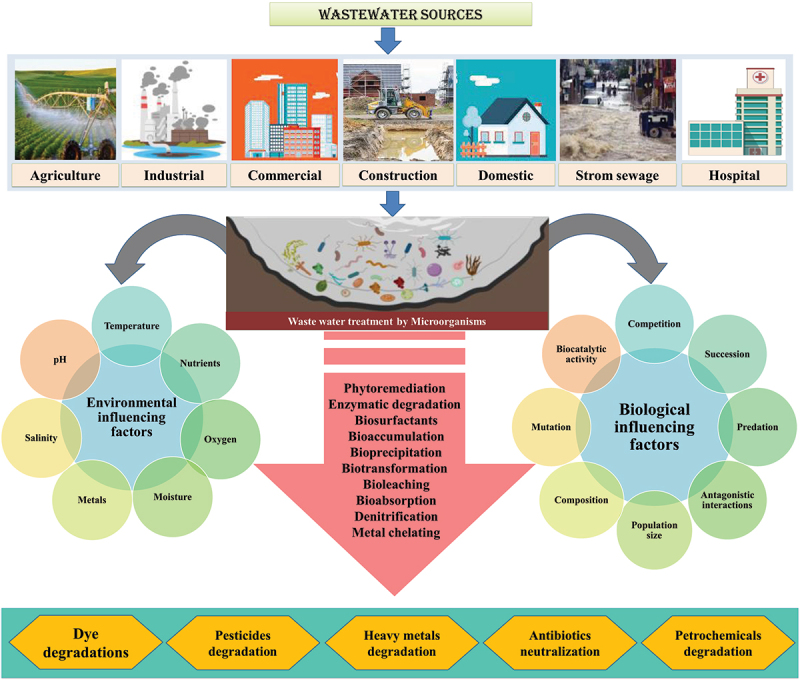
A schematic diagram of wastewater treatment by microorganisms with their different bioremediation mechanisms.

Fungi can help in the removal of pollutants (heavy metals) by increasing their bioavailability and converting them to lesser toxic forms [124]. Fungi are simple to grow and produce a significant amount of biomass. Several fungal strains have shown the ability to digest a variety of environmental contaminants, including dyes, pharmaceutical drugs, aromatic hydrocarbons, and heavy metals [125,126]. The two important characteristics of fungi that make them an ideal candidate for wastewater treatment are the secretion of many extracellular enzymes [127] and the hyphal mesh of fungi that protects the internal sensitive organelles from the ill effects of contaminants. Fungi are drawn to the rhizosphere by root exudates. Many factors influence plant-fungi interactions in the rhizosphere, including soil characteristics, plant species, water type, climate, and other microorganisms [128]. Plant-fungi interactions perform a variety of important functions, including metal-chelating siderophores emission, denitrification, and detoxification (Figure 3). The organic wastes are transformed into industrially important biochemicals and other valuable compounds by fungi, which is an advantage of using fungal culture in wastewater treatment over bacterial culture (proteins, organic acids). Animal feed can also be made from fungal biomass [129]. *Pleurotus pulmonarius, Stachybotrys sp., Cephalosporium aphidicola, Aspergillus parasitica, Verticillum terrestre, Candida sp., Acremonium sp., Glomus sp., Minimedusa sp., Talaromyces, Hydnobolites, Peziza*, and other fungal species can be used in wastewater treatment [130]. Table 6 shows the effective fungal degradation of wastewater.

**Table 6.** Some common fungal species used in pollutant remediation in wastewater.

Fungi	Wastewater type	Pollutant	Reference
*Penicillium, Saccharomyces*	Radioactive waste	Radionuclides U, Th, Sr	[176]
*Aspergillus niger, Rhizopus oryzae, Saccharomyces cerevisiae, Penicillium chrysogenum*	Industrial wastewater	Cr	[177]
*Candida sphaerica*	Industrial wastewater	Fe, Zn, Pb	[178]
*Candia sp.*	Industrial wastewater	Cu, Ni	[179]
*Candida porapsilosis*	Industrial wastewater	Hg	[180]
*Sphaerotilusnatans*	Industrial wastewater	Cr	[181]
*Gloeophyllumsepiarium*	Industrial wastewater	Cr	[182]
*Aspergillus niger*	Industrial wastewater	Fe	[183]
*X ray contrast agent iopromide and antibiotic ofloxacin*	Hospital wastewater	Trametesversicolor	[184]
*Trametesversicolor*	Veterinary hospital wastewater	Ciprofloxacin, trimethoprim, tetracycline, acridone, carbamazepine	[185]
*Trametes versicolor*	Urban wastewater	Salicylic acid, Codeine, Ceflalexine, acridone, ciprofloxacine, propanolol	[186]
*Trametes versicolor*	Pharmaceutical wastewater	Carbamazepine, benzophenone, diclofenac	[187]
*Pleurotusostreatus*	Hospital wastewater	Diclofenac, ketoprofen, atenolol	[188]
*Penicillumcorylophilum*	Domestic wastewater	Suspended solids	[189]

Over the last years, many research studies have suggested the effective role of ligninolytic fungi in degrading synthetic dyes [131]. Interestingly, fungi possess ligninolytic enzymes that degrade complex dyes, including laccase, manganese peroxidase, and lignin peroxidase. Some research studies reflecting fungi’s role in degrading dyes have been elucidated in Table 7.

**Table 7.** Shows the degradation of some dyes by fungi along with the mechanism involved.

Fungi	Dye	Mechanism	Reference
*Aspergillus flavus*	Malachite green	Biosorption and enzymatic degradation(Laccase and Manganese peroxidase)	[193]
*Aspergillus sp.,Pleurotus sp.*	Reactive Red-120	Biosorption and enzymatic degradation	[194]
*Aspergillus bombycis*	Reactive Red 31 dye	Biosorption and enzymatic degradation	[195]
*Aspergillus sp. TS-A (GMCC 12,964*	Mordant Yellow 1	Biosorption and enzymatic(Laccase degradation)	[196]
*Aspergillus niger*	Congo Red	Biosorption and degradation by fungal enzymes	[197]
*Cibacron Brilliant Red 3B-A*	Morchellaesculenta	Biosorption on fungal mycelium and biodegradation by Laccase enzyme	[198]
*Aspergillus salinarus*	Reactive Red HE7B	Biosorption and degradation by fungal enzymes	[199]
*Bjerkandera adusta*	Crystal violet Malachite Green Cotton Blue Methyl violet	Decolorization caused by the absorption of fungal biomass and enzymatic degradation	[200]

Many studies have shown that yeast can be used to eliminate pollutants (heavy metals) from the environment [132]. Yeast can also help reduce COD levels and remove mono and polyphenols [133] because yeast can absorb, accumulate, and degrade toxic compounds into nontoxic forms. It can be used to treat textile wastewater. *Saccharomyces cerevisiae, Galactomyces geotrichum*, *Trichosporon beigelii*, and *Candida krusei* can degrade dyes in wastewater [134].

### 4.3 Microalgae

These include the use of eukaryotic algae and cyanobacteria for biological wastewater treatment [135]. The term ‘phycoremediation’ refers to the use of algal species for bioremediation. *Chlorella* sp., *Picochlorum* sp., *Tetraselmis* sp., *Scenedesmus* sp., and other algal and cyanobacterial strains like *Anabaena* sp., *Oscillatoria* sp., *Spirulina* sp., *Chroococcus* sp., *Pseudospongiococcus* sp., *Scytonema* sp., *Dolichospermum* [136] are used in wastewater treatment. Microalgae have the following characteristics that make them an ideal candidate for wastewater treatment:

- Capability to utilize both inorganic and organic carbon, nitrogen, and phosphorus present in wastewater for growth [137].
- The life cycle is short and requires less nutrients [164].
- Scope of re-using algal biomass through adsorption/desorption mechanism [165].
- The growth of algal biomass is independent of environmental conditions, hence can be produced throughout the year [166].
- The efficiency of algal biomass is better than membranes to remove heavy metals [167].
- Source of Oxygen and helps in degradation process by heterotrophic bacteria [168].
- Useful in both anaerobic and aerobic effluent treatment plants [169].

Depending on the nutrient source, capital investment, and culture conditions (biofuels, CO_2_ capture), microalgae culture-based wastewater treatments can be open or closed.

#### 4.3.1 Open type

Algae are grown in open systems in places like ponds, lagoons, and deep channels. Natural (ponds, lagoons) or artificial (man-made ponds, tanks, containers) sites can be used. For domestic and industrial wastewater treatment, stabilization ponds containing bacteria and microalgae culture are most commonly used in temperate and tropical climates. Many studies have demonstrated the effective use of open microalgal cultured treatment plants in treating wastewater [170].

#### 4.3.2 Closed systems

Microalgae is grown in closed environments in such systems. Photobioreactors are one example of such a system. Reduced water evaporation, higher biomass yield, and contamination elimination are all advantages of closed-type treatment over open type [171]. A pilot-scale tubular bioreactors are used to grow a diverse range of microalgae, including *Arthrospira* sp., *Chlorella* sp., *Haematococcus* sp., *Spirulina* and *Phaeodactylum*sp [172].

Algal biosorbents have a high sorption capacity [173]. Using algae-based biosorption, heavy metal ion extraction from wastewater could be an environmentally friendly, cost-effective, and efficient method [174]. Textile wastewater contains algae cultivation nutrients (phosphates, nitrates, micronutrients, etc.) as well as organic dyes [175]. Many studies (Table 8) have shown that microalgae can remove pollutants from wastewater; for example, *C. vulgaris* and *S. quadricauda*can remove nitrate [190]; *Chlorella* sp., *Scenedesmus* sp., *Cosmarium*sp. for wastewater treatment [191]; *Chlorella* sp., *Scenedesmus* sp., *Cosmarium* sp. for treatment of wastewater (aquaculture wastewater and textile wastewater [192]. According to Ojha et al. [207], *C. vulgaris* and *S. quadricauda* cultures can be used for wastewater remediation.

**Table 8.** Some microalgae species used in wastewater treatment.

Wastewater type	Pollutant	Microalgae	Reference
Municipal wastewater	Organic waste	*Chlorella vulgaris*	[201]
Synthetic wastewater	Tetracycline	*Chlorella vulgaris*	[202]
Synthetic wastewater	17 α- Ethynylesteradiol	*Desmodesmussubspicatus*	[203]
Effluent from the wastewater treatment plant	Ciprofloxacin, Progesterone, paracetamol, diclofenac	*Chlorella pyrenoidosa*	[204]
Pharmaceutical industry wastewater	Salicylic acid, paracetamol	*Chlamydomonasmexicana*	[205]
Municipal wastewater	Organic waste	*Scenedesmus sp.*	[206]

Organic dyes are major pollutants in water. They can be found in many manufacturing industries, including textiles, plastics, and medicines. These dyes, when accumulated in aquatic systems, results into eutrophication and limited reoxygenation capacity. The production of poisonous amines during the decomposition of dyes is one of the most serious concerns [208]. Microalgae and Cyanobacteria represent a possible option for the bioremediation of wastewater. Microalgae decolorize dyes by adsorption or degradation. Microalgae can make use of wastewater dyes and nutrients. During the bioconversion process, microalgae can consume the dyes as a source of carbon and convert them to metabolites. The degradation of dyes by microalgae has been elucidated through some research studies in (Table 9).

**Table 9.** Shows the degradation of some dyes by microalgae along with the mechanism involved.

Microalgae	Dye	Mechanism	Reference
*Gonium sp.*	Reactive Blue 220	Absorption on algal biomass and enzymatic degradation	[213]
*Nostoc comminutum*	Remazol Black 5 Remazol Brilliant Blue	Absorption on algal biomass and enzymatic degradation	[214]
*Aspergillus terreus*	Direct Blue 1	Enzymatic degradation by Laccase and Manganese peroxidase	[215]
*Trametesgibbosa*	Reactive Black B	Enzymatic degradation by Manganese peroxidase, laccase	[216]
*Spirulina platensis*	Acid Black 210 Acid Blue 7 Reactive Black 5	Absorption on algal biomass and enzymatic degradation	[217]
*Spirulina*	Direct Yellow 12	Absorption on algal biomass and enzymatic degradation	[218]
*Chlorella vulgaris*	Reactive Black NN	Absorption on algal biomass and enzymatic degradation	[219]
*Nostoc carneum*	Methyl Orange	Absorption on algal biomass and enzymatic degradation	[220]
*Chlorella vulgaris*	Congo Red	Absorption on algal biomass and enzymatic degradation	[221]
*Chlorella pyrenoidosa*	Methylene Blue	Absorption on algal biomass and enzymatic degradation	[222]
*Candida tropicalis*	Acid Red B	Enzymatic degradation by Laccase and Lignin peroxidase	[223]

## 5 Factors affecting microbial biodegradation

Microbes can degrade various physical and chemical wastes through removal, alteration, immobilization, or detoxification. Microbes play a role because of their enzymatic pathways. Many factors influence bioremediation efficiency, including soil type, temperature, pH, oxygen, and other electron acceptors, nutrients, biological factors, and so on.

### 5.1 Environmental determinants

Temperature is the most important of all the physical factors that influence microorganism survival [209]. Microbial enzymes involved in biodegradation require the right temperature to metabolize substances. The rate of microbial activity increases as the temperature rises and peaks at the optimum temperature. The temperature of water influences various processes such as mineralization, diffusion, and chemical reactions [210]). Temperature extremes can kill bacteria and other microbes, affecting their growth [211]. Increases in temperature within the optimum range raise the reaction temperature, thereby increasing the solubility of contaminants, improving diffusion, and so on. The bacterial consortium of *Bacillus pumilus* HKG212 and *Zobellella taiwanensis*AT was used by [212] to degrade reactive green 19, and their findings revealed that the highest degradation occurs at 32.04°C.

The measurement of pH indicates microbial growth potential [224]. The pH range determines the survival of bacterial species, and thus bioremediation. Acidophilic, neutrophilic, and alkaliphilic biodegrading bacteria require acidic, neutral, and basic media for optimal activity [225]. According to [226], the pH of the affected site can be changed to achieve the desired biodegradation results. At pH 4.5, they were able to degrade malachite green by 98% using RuO_2_-TiO_2,_and Pt coated Ti mesh electrodes.

Moisture has an impact on the rate of biodegradation because moisture affects the content and concentration of soluble materials available, as well as the osmotic pressure and pH of aquatic systems.

Different microbes require different oxygen levels, such as aerobic, anaerobic, and semi-anaerobic conditions. In most cases, the presence of oxygen can help with hydrocarbon metabolism. Some contaminants, such as petroleum hydrocarbons in wastewater, inhibit bacterial growth by reducing compressed oxygen and electron acceptors [227]. Although shaking can improve oxygenation, delivering enough oxygen for the biodegradation of organic pollutants is a part of an operational issue and is costly [228].

An optimum quantity of nutrients and other chemicals is important for microbial metabolism [229]). Additional input of nutrients changes the nutrient balance for microbial growth, affecting the rate and effectiveness of biodegradation [230]. Microbes require various nutrients, including carbon, nitrogen, and phosphorus, to survive and continue their metabolic activities [231]. Varjani et al. [232] identified phosphorus as a critical factor in microbe growth.

#### 5.1.1 Contaminant concentration

The type and number of contaminants can have an impact on biodegradation. A high biodegradation rate can be achieved by increasing the contaminant concentration [233]. Heavy contaminants, such as oil petroleum-containing wastewater, have been fatal to the microbial community and negatively impact their biocatalytic activity. Low molecular weight contaminants with simple structures can achieve a high bioremediation rate [234]. Kerosene, for example, can be completely biodegraded at optimal concentrations due to its simple structure and low molecular weight [235].

#### 5.1.2 Salinity

According to [236] organic pollutants present in wastewater which contain alkaline chemicals biodegrade very slowly due to their ability to persist in waste. Contaminants with a high salt content may reduce biodegradation activity by inhibiting biological movement [237].

### 5.2 Biological factors

Biological factors influence the breakdown of organic pollutants as microorganisms compete for limited carbon sources, and antagonistic interactions between microbes exist. Major biological factors that affect the bioremediation activity of microbes include enzyme activity, interactions (competition, succession, and predation), population size and composition, mutation, etc. [238]. The rate of biodegradation is dependent on the substrate as well as the biocatalyst [239] and the specificity of the enzyme. Inhibition of enzymatic activities due to several factors like competition for carbon and nutrient sources can affect the biodegradative activity of microbes [240].

## 6 Microbial consortium: emerging technology

The microbial consortium is the emerging biotechnology-based green approach. Using a single microbe strain to treat wastewater may not give effective results, and efficiency can be compromised. Thus, many research findings have proposed applying microbial consortia [241,242]. Consortia comprising different groups of environmental microbes capable of degrading pollutants in wastewater can be an effective choice. Such consortiums have many advantages over the application of a single strain like fast removal, assistance in secondary application of treated wastewater, along with promoting ecological sustainability.

In the natural habitat, biofilm is formed by aggregating different groups of microbes attached through exopolymeric substances. The whole system is synergistic with microbial partners’ contributing toward forming a strong community [243].

The development of consortia is an emerging approach for wastewater treatment. The algal-bacterial consortium has many advantages owing to its biomass refiniability and reduced power consumption [244]. The fundamental principle in the microbial community is utilizing beneficial relationships which are promoting in pollutant removal from wastewater. The synergy is observed in the relationship wherein bacteria are involved in BOD removal, and algae remove nitrogen and phosphorus by absorption [245]. The relationship established between algae and bacteria provides a suitable ground for bioremediation [246]. Photosynthesis is undertaken by cyanobacteria bacteria and converts inorganic carbon present in wastewater to organic carbon [247]. The CO_2_ produced by bacterial oxidation serves as the carbon source for photosynthetic algae. Decomposers like *Acinetobacter* can remove BOD and oxidize organic carbon sources into CO_2,_ which serves in algae growth [248].

Extensive research findings have supported microbial consortium as a potential candidate for wastewater treatment [249]. Recent experimental studies conducted by [250] revealed the application of Ecobacter bacterial consortium facilitated the bioaugmentation for the biological removal of nitrogen compounds; showing ammonium was transformed by the microorganism reduction reaction; thus, presented decrease in the concentration of ammonium at the end of the treatment period. In their studies, Qi et al. [251] proved effectively that a well-established microbial (algal-bacterial) consortium in the phycosphere can be optimized and used in advanced wastewater treatment. The results of the research conducted by [252] showed that treatment of paper pulp wastewater by microbial consortium between microalgae and bacteria allowed good efficiency in removal of organic matter and nutrients. Rehman et al. [253] studied microbial consortium with *Klebsiella* sp. LCR187, *Bacillus subtilis* LOR166, *Acinetobacter* sp. BRS156 and *Acinetobacter junii* TYRH47 and *Typhadomingensis* and *Leptochloafusca*to treat oil field wastewater. Tara et al. [254] reported greater than 90% removal efficiency of pollutants from textile wastewater using microbial consortium. Leong et al. [255] reported 94% pollutant removal efficiency from municipal wastewater using microalgae consortium with bacteria. Microbes carry out the degradation through the secretion of various enzymes and organic acids [256]. Monica et al. [257] used Effective Microorganism (EM), which comprises *Lactobacillus*, *Aspergillus, Pseudomonas*, *Streptomyces*, and *Saccharomyces*, for biodegradation of sewage load in the water. *Lactobacillus* does the breakdown of lignin and cellulose in this consortium. *Pseudomonas* releases bioactive compounds which act on sewage and detoxifies or precipitate the metal. *Aspergillus* decomposes organic matter rapidly, producing alcohol and esters. Table 10 shows the effective utilization of consortia between microbes for treating wastewater from various sources.

**Table 10.** Microbial consortium for treatment of wastewater.

Consortium species	Genus	Complex wastewater from effluent treatment plant	Reference
Algal Concortium	*Chlorella sp*. *Nitzschia acicularis*	Reactor secondary waste water	[258]
Bacterial Consortium	*Methanosarcina sp*., *Methanotrix sp*., *Methanoculleus sp*., *Methanobacterium sp*., *Methanospirillum sp.*	Complex wastewater from Effluent treatment plant	[259]
Algal Consortium	*Chlorella vulgaris* *Scenedesmus sp*. *Westella botryoides*	Sewage waste water	[260]
Bacterial Consortium	*Acinetobacter junii, Rhodococcus sp*., *Pseudomonas indoloxydans*	Complex wastewater from Effluent treatment plant	[261]
Fungal Consortium	Arbuscular Mycorrhizal fungi with *Phragmites australis*	Complex wastewater from Effluent treatment plant	[262]
Fungal consortium	*Scedosporium apiospermum* and *Aspergillus orchraceus*	Complex wastewater from Effluent treatment plant	[263]
Bacterial consortium	*Lactobacillus, Saccharomyces, Aspergillus, Pseudomonas, Streptomyces*	Complex wastewater from Effluent treatment plant	[264]
Bacterial consortium	*Bacillus subtilis, Bacillus Thuringiensis, E. coli, Rhodopseudomonas palustris, Rhodobacter spheroids, Lactobacillus* sp.	Complex wastewater from Effluent treatment plant	[265]
Bacterial consortium	*Pseudomonas* sp, *Actinomyceta*sp, *Bacillus sp*., *Streptomyces sp*., *Staphylococcus sp.*	Complex wastewater from Effluent treatment plant	[266]
Bacterial consortium	*Vibrio, Staphylococcus, Aerococcus, Acinetobacter, Exiguobacterium*	Petrochemical wastewater	[267]
Fungal consortium	*Trametes versicolor, Irpexlacteus, Ganoderma lucidum, Phanerochaetechrysosporium*	Industrial wastewater	[268]
Fungal consortium	*Bjerkandera sp. R1*, *Bjerkanderaadusta, Phanerochaetechrysosporium*	Industrial wastewater	[269]
Fungi- Algae consortium	*Aspergillus niger (fungi); Chlorella vulgaris (algae)*	Pharmaceutical wastewater.	[270]
Algae	*Scenedesmus obliquus, Chlorella obliquus*	Pharmaceutical wastewater	[271]
Algae	*Lessonia nigrescens*, *Macrocystis integrifolia*	Complex wastewater from effluent treatment plant	[272]
Algae	*Anabaena cylindrica, Spirulina platensis* *Chlorella, Anabaena, Chlorococcus*	Synthetic wastewater	[272]
Algae	*Haematococcus pluvialis, Chlorella sp, Selenastrumcapricornutum*	Synthetic wastewater	[250]
Microalgae and Cyanobacteria	*Chlorella sp*. and*Phormidium sp.*	Tannery wastewater	[251]

## 7 Conclusion

Water contamination from various sources has become a serious problem around the world. The use of microbes as a treatment for water pollution is a viable alternative. Microbial remediation is an evolutionary and revolutionary technique for wastewater treatment that is currently in use. Microbes (bacteria, fungi, algae, and yeast) are naturally occurring and thus offer a long-term solution to the problem of water pollution. Lack of appropriate information about microbes’ metabolic capacity to degrade contaminants and a lack of controlled conditions such as temperature, pH, the appropriate number of contaminants, nutrients, and more time consumption are all possible limitations with the use of microbes in the treatment process. If the process is not controlled, contaminants may not be completely degraded, resulting in toxic byproducts. As a result, appropriate inside characterization can be an effective way to overcome the drawbacks of microbial-assisted wastewater remediation. Success in microbial wastewater treatment can also be attributed to advances in genetic engineering. Engineered microbial strains with high metabolic potential and well-understood detoxification pathways will undoubtedly aid in combating the wastewater threat to the greatest extent possible. Although several studies have been performed on the use of microbial consortium like microalgae-bacterial systems for the treatment of waste water, but still there is a need for further research in optimizing parameters for large-scale units. Maintaining the stability of the consortium is the main challenging task. More emphasis should be placed on some parameters viz. on selection of capable microbial strains, modeling the system in the long run and optimizing operational parameters, techno economic feasibility, etc. The present era demands to develop environmentally friendly technology that is also commercially viable. Engineered microbes must be integrated from the scientific stage to the practical and pilot stage in order to make significant advances in the use of microbes in the wastewater treatment process. Effective coordination across various disciplines and updated technologies are required to develop better environmental management in the near future.
